# The Spread of Infection from Small-Pox Hospitals

**Published:** 1902-03-22

**Authors:** 


### The Spread of Infection from Small-pox Hospitals.

The remarkable extent to which small-pox has prevailed during the past few months at Purfleet, a village situated on the northern banks of the Thames, just opposite the spot at which the small-pox ships are moored off the southern banks of the river, offers strong presumptive evidence in favour of the theory that the infection of small-pox can be conveyed for considerable distances through the air. Although the facts recently published by Dr. Thresh, Medical Officer of Health for the County of Essex, offer no proof of the theory in the slightest degree comparable with that formulated by Mr. Power a quarter of a century ago from a study of the Fulham outbreak, no one can doubt that what has happened will have the effect of greatly strengthening the opinion universally held by landholders, to the effect that a small-pox hospital is a nuisance to be kept at arms' length by every means which can be invented by legal ingenuity, and will thus add much to the difficulty, already great enough, of finding sites for these most necessary institutions. It seems that during the second quarter of last year only six cases of small-pox were notified in London, but that during the third quarter the disease spread so considerably that there were 272 cases. This increase in the number of acute cases in the small-pox ships was quickly followed by the presence of the disease in Purfleet, where cases occurred with such frequency that between the middle of September and the beginning of February about one-tenth of the whole population had been attacked. Nor was this merely a part of a widespread epidemic falling with equal virulence on other parts of the surrounding area, for, according to the calculations made by Dr. Thresh, the incidence of the disease on the population of Purfleet was far in excess of that on any other part of the district. Up to February 1st nearly four times as many cases, in proportion to population, had occurred in Purfleet as in the remainder of West Thurrock, and 30 times as many as in the rest of the Orsett Union, notwithstanding the fact that in another part of this union, namely at Grays, a considerable epidemic of small-pox (probably derived from Purfleet) had been in progress all the time.

Comparing the whole of the Orsett Union with the whole of Essex, we find that in proportion to the population there had been 40 times as many cases in the Orsett Union as in the remainder of the county, and if we then remember that in Purfleet the disease had been 30 times as prevalent as in the rest of the Orsett Union a very slight calculation will tell us how enormously excessive had been the prevalence of the disease in this little hamlet which lies opposite the metropolitan small-pox ships. After a careful consideration of the prevailing direction of the wind during the months in question and the condition as to vaccination of the population of Purfleet as compared with that of the rest of the district, Dr. Thresh has no hesitation in putting down the outbreak to the ships. We need hardly point out how serious a matter this is nor how much it is likely to add to the difficulties which are even now experienced in providing isolation accommodation. Some time ago the Local Government Board issued a memorandum on this subject in which they laid it down, "with a view of lessening the risk of infection, that a local authority should not contemplate the erection of a small-pox hospital—1st. On any site where it would have within a quarter of a mile of it as a centre either a hospital, whether for infectious diseases or not, or a workhouse or any similar establishment, or a population of 150 to 200 persons. 2nd. On any site where it would have, within half a mile of it as a centre, a population of 500 to 600 persons, whether in one or more institutions or in dwelling-houses." But the argument to be derived from the Purfleet outbreak goes far beyond this regulation, for Purfleet does not come within either the quarter-mile or the half-mile zone, but lies between half and three-quarters of a mile from the ships. Moreover, Dr. Thresh drives in his argument by quoting the prevalence of small pox at a village called Aveley, a place which is very much further away still. Indeed, he gives it as his impression that in the direction of the wind the infection of small-pox may be carried two, or possibly three, miles. It is evident that if we accept a theory we cannot draw a line just where we find it convenient to cease to apply it, and that if we are to believe that small-pox infection can drift across a river for nearly three-quarters of a mile, in such a virulent state of concentration as to attack 80 per cent. of the unvaccinated persons within a certain area during a period of seven months, as we are told has been the case, and that in certain directions of the wind it may "carry" even a couple of miles further, all our attempts to isolate the disease must of necessity be futile. We are left in the dilemma of having to admit either that the theory is wrong, or that a good deal of the money which we are spending in "isolating" small-pox patients is being wasted. In any case, the next time an owner of land prosecutes a sanitary authority for establishing a nuisance in the shape of a small-pox hospital in his neighbourhood, the lawyers will have a very interesting bit of evidence to bring before the court.

